# The Association between Dietary Intake of Folate and Physical Activity with Psychological Dimensions of Depressive Symptoms among Students from Iran

**DOI:** 10.1155/2013/582693

**Published:** 2013-11-14

**Authors:** Teymoor Yary

**Affiliations:** Department of Epidemiology, Faculty of Health, Ilam University of Medical Sciences, Ilam, Iran

## Abstract

Depression in students is a major public health problem. Although several risk factors associated with depression have been identified, the cause of depression is still not clear. Several studies have demonstrated that physical activity and nutrient intake, such as increased levels of B vitamins in serum, decrease symptoms of depression. The aim of this study was to investigate the association between physical activity and dietary intake of vitamins B_6_, B_9_, and B_12_ and symptoms of depression among postgraduate students. The results of this study suggest that intake of vitamin B_9_ may modulate the total score of Center for Epidemiological Studies Depression Scale (CES-D) and two subscales of the CES-D including depressive affect and interpersonal difficulties. This study also showed that moderate/high levels of physical activity were inversely and significantly associated with symptoms of depression (total scores) and three subscales of the CES-D including depressive affect, positive affect, and somatic complaints.

## 1. Background

Depression has increasingly become a public health problem in both developed [1] and developing countries [2]. Several risk factors cluster together and increase the risk of depressive disorders. Nonmodifiable, modifiable, and contextual risk factors are causes of these disorders. Advancing age, gender [3], and ethnicity [4] are examples of nonmodifiable risk factors of depression. Inflammation [5], cigarette smoking, physical inactivity, poor nutrition, and consumption of alcohol are modifiable risk factors of depression [6]. Sociodemographic variables such as education, income, health insurance, and poverty are contextual risk factors of depression. Accumulations of one or more risk factors in individuals may lead to depressive disorders and will become a public health problem when these people become unable to conduct normal activities of daily living or cause distress to other healthy people in the society.

Depressive disorders have been shown to be associated with the higher rates of mortality compared with individuals without depressive disorders [7]. These disorders have negative effects on the quality of life and decreased life expectancy, especially among the severely mentally ill [8]. It has been shown that depression has a negative impact on many diseases such as cancer [9], cardiovascular disease [2], and diabetes mellitus [10]. A significant elevation in morbidity and mortality has been shown in these diseases because of depression [2, 9, 10].

Symptoms of depression among college students are a growing public health concern [11–13]. One out of seven students may experience depression, which usually presents as feelings of fatigue, guilt, sadness, and hopelessness. Depressed students are prone to low academic performance, withdraw from university, increased smoking, acute infection illnesses, self-injurious behavior, and suicide [14]. Depression may interfere with interpersonal relationships and performance of daily tasks, which may lead to suicidal thoughts and attempts of suicide [15].

Depression in college students may be due to several factors, including vulnerable age, demands of college life, personal issues, adaptation to a new environment, tendency toward perfectionism, and conflict between traditional and modern values [15, 16]. There is increasing interest in the role of physical activity in the prevention and treatment of depression [17]. Over the last 40 years, several studies have investigated the relationship between physical activity and improvements in mental health symptoms among various populations. Many of the studies have indicated that physical activity is able to reduce the risk of several mental health conditions, especially depressive symptoms [18–20]. Numerous gaps remain in each of these individual research areas. For example, these studies have been limited by lack of data on important factors such as levels of physical activity, because although previous studies have reported that physical activity successfully decreases depression, it is not clear which levels of physical activity were related to depression [21]. Furthermore, the role of physical activity as a predictor of depression was not constant [22].

An association between depressive symptoms and polyunsaturated fatty acids or minerals has been found among university students [2, 12, 23, 24]. Also, a few studies among other populations have demonstrated that dietary intake of vitamins B may decrease symptoms of depression [25–27]. However, these studies have been limited in middle age and over, and the association between dietary intake of vitamin B such as vitamin B_9_ has not received as much attention in special populations such as university students. Also, studies have investigated the association between physical activity and depression [28, 29]. These studies failed to control nutrients intake such as vitamins B, which may reduce symptoms of depression, and this can confound the association between physical activity and depression. In addition, several previous studies did not investigate the relationship between the levels of physical activity with depression. The current study excluded several other confounders such as hyperthyroidism, hypertension, diabetes, cancer, or heart disease; these diseases can affect the associations between vitamins B and both depression and physical activity; these confounders cannot be controlled statistically utterly. Most importantly, there is no any data (locally or universally) on the subject of vitamins B and depression and psychological dimensions of depression in university students. Taking everything into consideration, the association between vitamins B/physical activity and depressive symptoms remains unclear among university students. Therefore, the aim of this study was to investigate the association between physical activity and dietary intake of vitamins B_6_, B_9_, and B_12_ and symptoms of depression and psychological dimensions of depression as measured by the CESD subscales (depressive affect, somatic complaints, positive affect, and interpersonal difficulties).

## 2. Materials and Methods

A cross-sectional study, designed in 2011, was performed on a convenience sample of 425 Iranian students who were studying in Malaysia, aged 32.54 ± 6.19 years. Based on the study design, students with serious diseases such as hyperthyroidism, hypertension, diabetes, or heart disease were excluded since such conditions may affect lifestyle (e.g., physical activity) or alter the risk factors for depression. Students who had a history of mental illness or those taking psychiatric drugs were also excluded. Consequently, 23 individuals were dropped from the study; data analyses were performed on the sample of 402 participants that remained. This study was approved by The Scientific Counselor and Director of Iranian Students Affairs in South East Asia in Malaysia, and informed consent was obtained from all participants before enrollment.

### 2.1. Depression Questionnaire

Symptoms of depression in our population were assessed with the Center for Epidemiologic Studies (CES-D) questionnaire [30]. Symptoms calculated with the CES-D included feelings of loneliness, appetite loss, sadness, sleep disorders, fear, and crying. This questionnaire contained twenty self-administered items, using a 4-point Likert-type scale that ranged from 0 (rarely or none of the time; less than 1 day) to 3 (most or all of the time; 5–7 days). Scores on the CES-D could range from 0 to 60. A cut-off score of 16 or greater indicated symptoms of depression. This questionnaire has been categorized in four dimensions including depressive affect, positive affect, somatic complaints, and interpersonal difficulties [30].

### 2.2. Assessment of Dietary B Vitamins

Dietary intake of B vitamins, including B_6_, B_9_, and B_12_, was assessed with a semiquantitative food frequency questionnaire (FFQ) [31]. The FFQ has been validated in several multiethnic populations [32, 33] and is used to assess food consumption over the previous 12 months. Nutritionist IV software, version 3.5.2, was used to measure the amount of B vitamins consumed. Univariate analyses were performed with SPSS software to analyze the relationships between the levels of B_6_, B_9_, and B_12_ calculated from the FFQ and the socioeconomic status and lifestyle factors of the participants.

### 2.3. Physical Activity Questionnaire

In the present study, physical activity was defined as leisure-time physical activity [34]. This questionnaire was used to investigate the activities the students performed during the last year, including transport to and from work during the last year. The questionnaire has a 4-point interview format or self-report items that measure physical activity. These items include 1 for almost completely inactive or low physical activity less than 2 h per week, 2 for low physical activity performed for 2–4 h per week, 3 for low physical activity intended for more than 4 h per week or more vigorous activity in favor of 2–4 h per week, and 4 for additional vigorous physical activity for over 4 h per week or regular heavy exercise or competitive sports many times per week. The final decision on physical activity was graded as follows: category 1 for low level of physical activity, category 2 for moderate level of physical activity, and categories 3 and 4 for high level of physical activity [34].

### 2.4. Statistical Analysis

Independent *t*-test was used to examine the associations between participant characteristics and symptoms of depression. To assess the association between depression and dietary intake of B vitamins, we generated a multiple linear regression model. In this model, depressive symptom was the dependent variable whereas vitamins B intake and other variables were the independent factors.

## 3. Results

Table 1 shows the characteristics of study participants based on total depression score (CES-D) and vitamins B. Symptoms of depression were significantly associated with decreased dietary reference intake (DRI) of vitamin B_9_ and age. Female gender was associated with higher score of depressive symptoms and more consumption of vitamin B_6_ and vitamin B_9_ compared to males. Less education was also associated with depressive symptoms and decreased dietary intake of vitamin B_9_. Marital status, current smoking, former smoking, body mass index (BMI), and number of close friends were not associated with symptoms of depression or vitamins B. Monthly expenses were significantly associated with less consumption of vitamins B but not with depressive symptoms. Living in campus was not associated with depressive symptoms or the intake of vitamins B. A high level of depressive symptom was associated with low levels of physical activity, independent of vitamins B.

**Figure 1:**
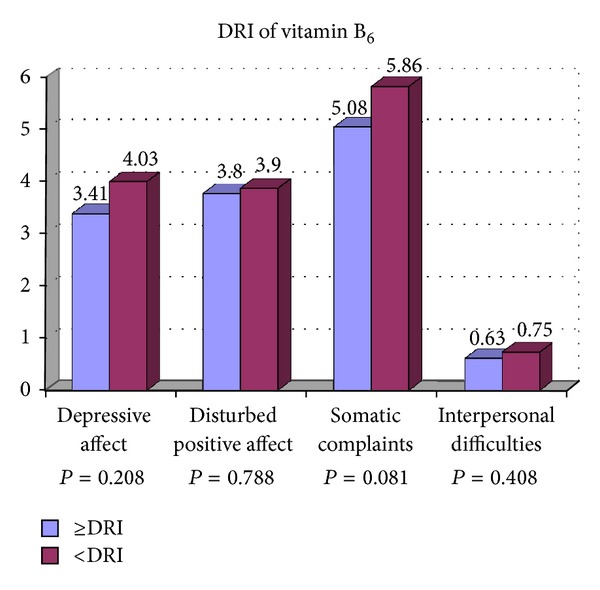
The association between vitamin B_6_ and psychological dimensions of depression.

**Figure 2:**
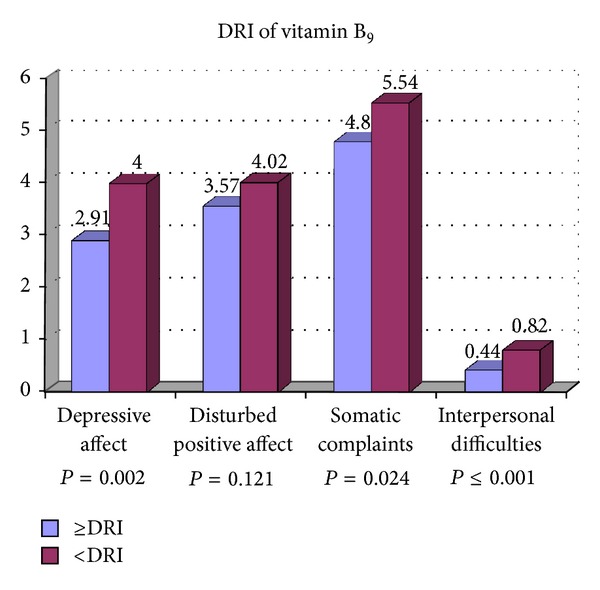
The association between vitamin B_9_ and psychological dimensions of depression.

**Figure 3:**
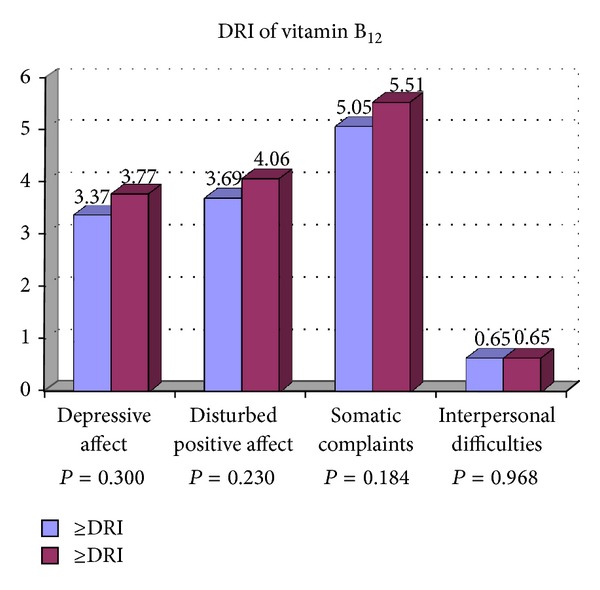
The association between vitamin B_12_ and psychological dimensions of depression.

**Figure 4:**
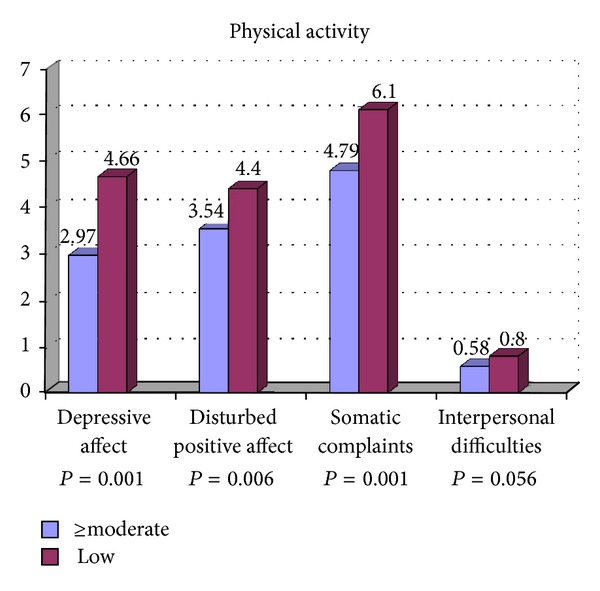
The association between physical activity and psychological dimensions of depression.

Table 2 presents the associations between the participants' characteristics, DRI of vitamins B_6_, B_9_, and B_12_ with the psychological dimensions of depression including depressive affect, somatic complaints, positive affect, and interpersonal difficulties. Less consumption of vitamin B_9_ was significantly associated with depressive affect and interpersonal difficulties compared with participants who consumed more vitamin B_9_. The psychological dimensions of depression were not related to current, past smoking, marital status, monthly expenses, and DRI of vitamins B_6_ and B_12_. Postgraduate students with lower education had a significant higher score of depressive affect and interpersonal difficulties. Female gender was significantly associated with depressive affect than males. Higher score of depressive affect was significantly associated with participant who had lower age. Lower BMI was also associated with higher score of depressive affect. Participant with less than five close friends had higher score of depressive affect and interpersonal difficulties compared to those with more close friends. Higher score of depressive affect, somatic complaints, and positive affect was associated with low level of physical activity. Living in campus was significantly associated with interpersonal difficulties compared to those who were living out of campus. Figures 1, 2, 3, and 4 show the association between vitamins B_6_, B_9_, and B_12_ and physical activity with psychological dimensions of depression.

Total score of the CES-D was not associated with DRI of vitamins B_6_ and B_12_. The score of CES-D was higher in participants who consumed fewer vitamin B_9_ compared with participants who consumed more vitamin B_9_ (Table 3). This relationship total score of the CES-D and physical activity and vitamin B_9_ remained even after accounting for potential confounding variables such as age and sex (Table 4).

We conducted a multiple linear regression model analysis to identify the association between physical activity and dietary intake of B vitamins and the psychological dimensions of depression including depressive affect, somatic complaints, positive affect, and interpersonal difficulties. In this model, an inverse association between DRI of vitamin B_9_ and depressive affect, and interpersonal difficulties was found after adjusting for potential confounds, including sex, age, BMI, monthly expenses, close friends, living in campus, smoking, physical inactivity, education, marital status, and vitamins B_6_ and B_12_. This study also showed that moderate/high levels of physical activity were associated with symptoms of depression (total scores) and three subscales of the CES-D including depressive affect, positive affect, and somatic complaints (Table 5).

## 4. Discussion

The present cross-sectional study determined that inverse relationships existed between intake of vitamin B_9_ and the total score of CES-D and two subscales of the CES-D-score including depressive affect and interpersonal difficulties among university students. This study also showed that moderate/high levels of physical activity were inversely and significantly associated with symptoms of depression (total scores) and three subscales of the CES-D including depressive affect, positive affect, and somatic complaints. The associations persisted even after adjusting for sex, age, BMI, monthly expenses, close friends, living in campus, smoking (current and former), education, marital status, and vitamins B_6_ and B_12_.

To the best our knowledge this is the first study to show an association between physical activity and vitamin B_9_ intake and the psychological dimensions of depression among Iranian university students. Our results and methodology used in this cross-sectional study is novel and unique because no study has investigated the association between depression/the psychological dimensions of depression and vitamins B and the levels of physical activity among university students.

An inverse association between depressive symptoms and vitamin B_9_ among our population can be supported by previous cross-sectional studies. A cross-sectional study documented that low intake of vitamin B_9_ was inversely associated with depression among currently smoking men and men with low anxiety levels [35]. A second cross-sectional study also showed a significant association between lower depressive symptoms and higher dietary intakes of vitamin B_9_ in middle-aged (42–60 y) men; the association was not found for other vitamins such as vitamins B_6_ and B_12_ [36]. A third cross-sectional study found an inverse association between vitamin B_9_ intake and prevalence of depression among men as well as an inverse association between depression and vitamin B_12_ among women; mean age was 41 and 34 years for men and women, respectively [37]. Conversely, the fourth cross-sectional study in men, aged 70–90 years, does not detected a relationship between symptoms of depression and vitamins B_6_, B_9_, and B_12_ [38]. Despite the results described above, it has been documented that vitamins B_6_ and B_12_ are involved in the synthesis of monoamines neurotransmitters in the central nervous system, such as dopamine, serotonin, norepinephrine, and epinephrine [39, 40].

The mechanism underlying the relationship between depression and vitamin B_9_ is unknown, but it can partly explain the role of vitamin B_9_ in the regulation of homocysteine. It was found that hyperhomocysteinemia is associated with depressive disorders [41]. For instance, research showed that more than 50% of depressed patients have hyperhomocysteinemia [42]. Homocysteine can increase oxidative stress, apoptosis, and DNA strand breakage; it is also directly toxic to neurons and blood vessels [43], and the vascular system of depressed patients may be destroyed by homocysteine. Three vitamins including B_6_, B_9_, and B_12_ are involved in the regulation of homocysteine [43, 44]. However, it has been indicated that hyperhomocysteinemia was more common in patients with vitamin B_9_ deficiencies [45]. One possible explanation involves dysfunction of metabolic pathways that require S-adenosylmethionine (SAM), which serves as a methyl donor in numerous biochemical processes including those important for neurological function. Under homeostatic conditions, SAM is formed from methionine but is subsequently metabolized to homocysteine after serving as a methyl donor. Vitamin B_9_ is required to donate one-carbon groups to homocysteine to recycle it to methionine for continued SAM formation. Deficiencies in vitamin B_9_ lead to accumulation of homocysteine, decreased SAM, and therefore impairment of neurological function [46–49].

Vitamin B_9_ deficiency may increase symptoms of depression by impairing the synthesis of tetrahydrobiopterin (BH4). BH4 is a compulsory cofactor for the three aromatic amino acids (tryptophan, phenylalanine, and tyrosine) hydroxylase enzymes. The three aromatic amino acids produce several neurotransmitters including serotonin (5-hydroxytryptamine, 5-TH), dopamine, melatonin, norepinephrine, and epinephrine [50]. Deregulation of this reaction may lead to abnormalities in the synthesis of mentioned neurotransmitters [51, 52], as well as depression [50].

The present study found an inverse association between depressive symptoms and moderate/high levels of physical activity among Iranian university students. Previous cross-several studies have shown that less depressive symptoms are associated with physically active individuals [28, 29]. The dose-response correlation between differing levels of physical activity and depression symptoms is not very clear. In only a few studies, individuals were categorized according to their level of physical activity into 2 or more groups to investigate a dose gradient relationship with depressive symptoms. A cross-sectional study conducted on adolescents found that total amount of physical activity was associated with symptoms of depression; however, moderate and vigorous physical activity was not independently related to depressive symptoms [28]. In contrast, a cross-sectional study concluded that high levels of physical activity were associated with less depression as compared to low level of physical activity [53].

There are several proposed mechanisms for the relationship between physical activity and depression symptoms. The effects of physical activity on depression mechanisms can be explained with both psychological and physiological/biochemical theories. It is to be expected that a combination of mechanisms influences the link between physical activity and depression [54]. Several factors related to depressive symptoms such as the sense of enjoyment, fulfillment, and social interactions can be provided by physical activities during leisure time [55]. One of the most frequently proposed mechanisms for the antidepressant effect of physical activity is cognitive-behavioral hypothesis [56]. The hypothesis suggests that negative feelings that may cause depression and activity releases may block these conditions. Physical activity seems to increase skill mastery, self-efficacy, feelings of success, and locus of control, and a lack of these factors is associated with depression symptoms [54]. Also, the time out/distraction hypothesis suggests that physical activity helps individuals overcome their daily worries and thus reduces depression symptoms [54, 56].

The effect of physical activity as an antidepressant can be further explained by the amine hypothesis. Individuals with depression have lower levels of monoamine neurotransmitters, including serotonin [57], dopamine, and norepinephrine [58]. Exercise can increase the level of these neurotransmitters [54]; this hypothesis is supported by antidepressant drugs such as tricyclic monoamine oxidase inhibitors and electroconvulsive therapy, all of which raise the level of amine transmission [56]. Furthermore, according to the endorphin hypothesis, exercise influences depression with an increased discharge of *β*-endorphins. Endorphins have a positive effect on mood and improve sense of happiness [59].

Physical activity can also modify several risk factors related to depressive symptoms. Many biological risk factors such as glucose intolerance, inflammation, and vascular dysfunction can be improved by physical activity; these factors have shown to trigger possibly important mechanisms leading to depression [60].

Our study found that women have higher levels of depressive symptoms as well as high consumption of vitamins B_6_ and B_9_ compared to men. A number of studies have shown that women have higher prevalence of depression versus men [56, 61]; however, the mechanism remains unclear. Researcher are trying to explain these differences through biological, psychological, genetic, and social factors [62, 63]. Higher levels of two psychological factors including interpersonal orientation and rumination among women were shown to be associated with the higher levels of depression [63]. Also, it has been suggested that higher rates of sexual harassment, poverty, chronic strain, and child abuse among females compared to men may cause of higher level of depressive symptoms [63]. In the matter of biological factors, the serotoninergic activity in the brain can be changed with female gonadal hormones, although the mechanism is not clear, but it is possible that gonadal hormones alter the regulation of the level of monoamines such as serotonin [64].

## 5. Limitation of Study

We controlled for several important variables linked with depression that may confound interpretation of the data, including sex, age, BMI, monthly expenses, close friends, living in campus, smoking habit, education, physical activity, and marital status. However, this study was a cross-sectional design, and therefore causality of dietary intake of vitamin B_9_ or physical activity and depressive symptoms cannot be determined.

## 6. Conclusion

The results of this study suggest that physical activity and intake of vitamin B_9 _ may modulate the total score of CES-D and psychological dimensions of depressive symptoms in university students.

## Figures and Tables

**Table 1:** Characteristics of the study subjects based on overall CES-D score and vitamins B.

Variables	Depression score (Mean ± SD)	*P*	*Vitamin B_6_ (Mean ± SD)	*P*	**Vitamin B_9_ (Mean ± SD)	*P*	**Vitamin B_12_ (Mean ± SD)	*P*
Age groups		0.010		0.309		0.030		0.484
≤35 (*n* = 284)	13.85 ± 9.22		2.48 ± 1.71		436.47 ± 352.53		3.62 ± 2.96	
>35 (*n* = 118)	11.59 ± 7.37		2.66 ± 1.25		526.52 ± 432.16		3.90 ± 5.18	
Gender		0.031		0.008		0.004		0.178
Female (*n* = 173)	14.27 ± 9.46		2.79 ± 1.91		529.98 ± 478.82		4.00 ± 4.70	
Male (*n* = 229)	12.37 ± 8.13		2.34 ± 1.32		412.23 ± 272.25		3.48 ± 2.81	
Education		0.001		0.068		0.031		0.295
<20 y (*n* = 199)	14.60 ± 9.23		2.38 ± 1.58		421.66 ± 344.75		3.50 ± 2.32	
≥20 y (*n* = 203)	11.80 ± 8.08		2.68 ± 1.64		503.33 ± 407.21		3.89 ± 4.74	
Marital status		0.289		0.323		0.111		0.855
Married (*n* = 208)	12.74 ± 7.82		2.61 ± 1.38		492.04 ± 358.89		3.73 ± 4.03	
Single (*n* = 194)	13.66 ± 9.68		2.45 ± 1.83		431.66 ± 398.64		3.66 ± 3.43	
Current smoking		0.700		0.875		0.711		0.235
Yes (*n* = 36)	13.13 ± 8.82		2.54 ± 1.64		465.10 ± 388.69		3.63 ± 3.53	
No (*n* = 366)	13.72 ± 8.28		2.49 ± 1.36		440.51 ± 269.16		4.41 ± 5.51	
Former smoking		0.338		0.356		0.327		0.860
Yes (*n* = 16)	13.27 ± 8.79		2.55 ± 1.64		466.68 ± 385.22		3.71 ± 3.80	
No (*n* = 386)	11.13 ± 8.26		2.17 ± 0.95		371.73 ± 168.94		3.54 ± 2.17	
BMI		0.084		0.347		0.919		0.635
≤25 (*n* = 262)	13.74 ± 8.96		2.48 ± 1.67		464.31 ± 424.30		3.77 ± 4.42	
>25 (*n* = 140)	12.15 ± 8.34		2.64 ± 1.45		460.27 ± 277.56		3.58 ± 1.95	
Monthly expenses ($)		0.862		0.014		0.009		0.020
<800 (*n* = 199)	13.10 ± 8.85		2.33 ± 1.65		411.68 ± 349.97		3.25 ± 2.28	
≥800 (*n* = 210)	13.26 ± 8.71		2.72 ± 1.56		509.73 ± 399.41		4.12 ± 4.69	
Close friends		0.077		0.117		0.122		0.263
<5 (*n* = 220)	13.89 ± 9.03		2.42 ± 1.67		436.25 ± 370.16		3.51 ± 3.07	
≥5 (*n* = 182)	12.34 ± 8.38		2.67 ± 1.54		495.12 ± 388.69		3.93 ± 4.42	
Physical activity		≤0.001		0.485		0.969		0.206
≥Moderate (*n* = 274)	11.89 ± 8.06		2.57 ± 1.71		462.40 ± 366.16		3.49 ± 2.38	
Low (*n* = 128)	15.95 ± 9.58		2.45 ± 1.39		463.97 ± 407.56		4.15 ± 5.64	
Living in campus		0.155		0.920		0.467		0.070
Yes (*n* = 170)	13.91 ± 9.58		2.52 ± 1.62		478.99 ± 436.08		3.38 ± 2.05	
No (*n* = 232)	12.65 ± 8.10		2.54 ± 1.61		451.11 ± 332.14		4.14 ± 5.22	

*mg/day; **μg/day.

**Table 2:** The association between vitamins B and characteristics of the subjects and psychological dimensions of depression.

Variables	Depressive affect (Mean ± SD)	P	Disturbed positive affect (Mean ± SD)	P	Somatic complaints (Mean ± SD)	P	Interpersonal difficulties (Mean ± SD)	*P*
DRI of vitamin B_6_		0.208		0.788		0.081		0.408
≥DRI (*n* = 338)	3.41 ± 3.56		3.80 ± 2.89		5.08 ± 3.14		0.63 ± 1.05	
<DRI (*n* = 64)	4.03 ± 3.85		3.90 ± 3.09		5.86 ± 3.73		0.75 ± 1.10	
DRI of vitamin B_9_		0.002		0.121		0.024		≤0.001
≥DRI (*n* = 183)	2.91 ± 2.99		3.57 ± 2.58		4.80 ± 2.86		0.44 ± 0.89	
<DRI (*n* = 219)	4.00 ± 3.99		4.02 ± 2.97		5.54 ± 3.51		0.82 ± 1.16	
DRI of vitamin B_12_		0.300		0.230		0.184		0.968
≥DRI (*n* = 264)	3.37 ± 3.67		3.69 ± 2.90		5.05 ± 3.15		0.65 ± 1.13	
<DRI (*n* = 138)	3.77 ± 3.47		4.06 ± 2.97		5.51 ± 3.42		0.65 ± 0.93	
Age groups		0.001		0.178		0.065		0.375
≤35 (*n* = 284)	3.83 ± 3.84		3.94 ± 2.96		5.38 ± 3.41		0.68 ± 1.11	
>35 (*n* = 118)	2.72 ± 2.83		3.52 ± 2.82		4.78 ± 2.80		0.58 ± 0.95	
Gender		≤0.001		0.092		0.996		0.311
Male (*n* = 229)	2.95 ± 3.21		3.60 ± 2.82		5.21 ± 3.16		0.60 ± 1.02	
Female (*n* = 173)	4.25 ± 3.26		4.10 ± 3.04		5.21 ± 3.38		0.71 ± 1.11	
Education		≤0.001		0.053		0.051		0.007
<20 y (*n* = 199)	4.18 ± 3.76		4.10 ± 2.88		5.53 ± 3.94		0.79 ± 1.18	
≥20 y (*n* = 203)	2.86 ± 3.34		3.54 ± 2.95		4.90 ± 3.08		0.51 ± 0.91	
Marital status		0.051		0.651		0.553		0.134
Married (*n* = 208)	3.17 ± 3.17		3.88 ± 2.85		5.12 ± 2.96		0.57 ± 0.94	
Single (*n* = 194)	3.88 ± 4.00		3.74 ± 3.00		5.31 ± 3.54		0.73 ± 1.17	
Current smoking		0.676		0.607		0.980		0.789
Yes (*n* = 36)	3.75 ± 2.72		4.06 ± 3.10		5.22 ± 3.23		0.69 ± 1.14	
No (*n* = 366)	3.49 ± 3.69		3.79 ± 2.91		5.21 ± 3.26		0.64 ± 1.05	
Former smoking		0.225		0.255		0.979		0.567
Yes (*n* = 16)	2.44 ± 3.24		3.00 ± 3.31		5.19 ± 2.37		0.50 ± 1.10	
No (*n* = 386)	3.55 ± 3.62		3.85 ± 2.91		5.21 ± 3.28		0.66 ± 1.06	
BMI		0.006		0.469		0.455		0.437
≤25 (*n* = 262)	3.87 ± 3.82		3.89 ± 2.97		5.30 ± 3.40		0.68 ± 1.07	
>25 (*n* = 140)	2.84 ± 3.30		3.67 ± 3.02		5.05 ± 2.95		0.59 ± 1.04	
Monthly expenses		0.780		0.211		0.445		0.196
<800 (*n* = 199)	3.56 ± 3.62		3.63 ± 2.75		5.34 ± 3.31		0.58 ± 0.97	
≥800 (*n* = 210)	3.46 ± 3.60		3.99 ± 3.07		5.10 ± 3.19		0.71 ± 1.14	
Close friends		0.020		0.187		0.853		0.009
<5 (*n* = 220)	3.89 ± 3.81		4.00 ± 3.03		5.24 ± 3.31		0.77 ± 1.15	
≥5 (*n* = 182)	3.05 ± 3.29		3.60 ± 2.78		5.18 ± 3.19		0.50 ± 0.93	
Physical activity		≤0.001		0.006		<0.001		0.056
≥Moderate (*n* = 274)	2.97 ± 3.31		3.54 ± 2.81		4.79 ± 3.06		0.58 ± 1.01	
Low (*n* = 128)	4.66 ± 3.95		4.40 ± 3.08		6.10 ± 3.47		0.80 ± 1.15	
Living in campus		0.340		0.964		0.052		0.026
Yes (*n* = 170)	3.72 ± 4.05		3.82 ± 2.74		5.58 ± 3.49		0.79 ± 1.24	
No (*n* = 232)	3.36 ± 4.24		3.81 ± 3.05		4.94 ± 3.04		0.54 ± 0.89	

DRI for vitamin B_6_: 19–50 y = 1.3 mg/d, >50 y = 1.7 mg/d; DRI for vitamin B_9_: 400 *μ*g/d; DRI for vitamin B_12_: 2.4 *μ*/d (DRI 1998).

**Table 3:** Association between vitamins B and overall CES-D score.

Variables	DRI of vitamin B_6_		*P*	DRI of vitamin B_9_		P	DRI of vitamin B_12_		P
	≥DRI (*N* = 338)	<DRI (*N* = 64)		≥DRI (*N* = 183)	<DRI (*N* = 219)		≥DRI (*N* = 264)	<DRI (*N* = 138)	
The score of CES-D (Mean ± SD)	12.93 ± 8.56	14.55 ± 9.76	0.175	11.73 ± 7.24	14.40 ± 9.72	0.002	12.77 ± 8.75	13.96 ± 8.77	0.186

**Table 4:** Association between vitamins B intake and physical activity and depressive symptoms in a regression model.

Variables	β	t	p
DRI of vitamin B_9_ (≥DRI versus <DRI) Physical activity (≥moderate versus low)	−0.14 −0.21	2.48 4.34	0.014 ≤0.001

Adjusted for sex (male, female), age (continuous), BMI (continuous), monthly expenses (continuous), close friends (continuous), living in campus (yes versus no), smoking (yes versus no), education (continuous), marital status (yes versus no), and vitamins B_6_ (continuous) and B_12_ (continuous).

**Table 5:** The association between DRI of vitamin B_9_ and physical activity and psychological dimensions of depression in a regression model.

Variables	Depressive affect			Disturbed positive affect			Somatic complaints			Interpersonal difficulties		
	*β*	*t*	*p*	*β*	*t*	*p*	*β*	*t*	*p*	*β*	*t*	*p*
Vitamin B_9_	−0.13	2.48	0.014	−0.07	1.18	0.239	−0.08	1.40	0.163	−0.21	3.83	≤0.001
Physical activity	−0.22	4.51	≤0.001	−0.12	2.43	0.016	−0.19	3.74	≤0.001	−0.10	1.95	0.052

Adjusted for sex (male, female), age (continuous), BMI (continuous), monthly expenses (continuous), close friends (continuous), living in campus (yes versus no), smoking (yes versus no), education (continuous), marital status (yes versus no), vitamin B_6_ (continuous), vitamin B_12_ (continuous), vitamin B_9_ (≥DRI versus <DRI), and physical activity (≥moderate versus low).
